# Robot-assisted orthopedic surgery in the treatment of adult degenerative scoliosis: a preliminary clinical report

**DOI:** 10.1186/s13018-020-01796-2

**Published:** 2020-07-25

**Authors:** Xiuyuan Chen, Fan Feng, Xiaosheng Yu, Shurong Wang, Zhipeng Tu, Yingchao Han, Quan Li, Hao Chen, Zhi Chen, Lifeng Lao, Hongxing Shen

**Affiliations:** Department of Spine Surgery, Renji Hospital, School of Medicine, Shanghai Jiaotong University, 160 Pujian Road, Shanghai, 200127 China

**Keywords:** Robot, Adult, Scoliosis, Pedicle screw, Internal fixators

## Abstract

**Study design:**

A single-institution, retrospective cohort study.

**Objective:**

To compare the accuracy and short-term clinical outcomes of pedicle screw placement between robot-assisted (RA) and freehand (FH) technique in the treatment of adult degenerative scoliosis (ADS).

**Methods:**

From February 2018 to October 2019, 97 adult patients with degenerative scoliosis admitted to our department were retrospectively reviewed. Thirty-one patients received robot-assisted pedicle screw placement (RA group), and 66 patients underwent freehand pedicle screw placement (FH group). Patient demographics and short-term clinical outcomes were recorded and compared between two groups. Gertzbein-Robbins grading system was adopted to evaluate the accuracy of pedicle screw placement by means of postoperative CT scan. Short-term clinical outcomes consist of operative time, intraoperative blood loss, length of hospital stay (LOS), radiological parameters, Scoliosis Research Society-22 (SRS-22) scores before the operation, 6 months after operation, adverse events, and revisions.

**Results:**

The accuracy of screw placement was higher than that of the FH group (clinically acceptable 98.7% vs. 92.2%; *P*< 0.001). Intraoperative blood loss of the RA group was less than those in the FH group (499 vs. 573 ml; *P* < 0.001). Operative time (283.1 vs. 291.9 min; *P* = 0.31) and length of stay (12.8 vs. 13.7 days; *P* = 0.36) were compared between RA and FH groups. In terms of radiological parameters, both of groups were improved postoperatively. The SRS-22 scores at 6 months after operation from both groups were better than those before operation. For surgery-related complication, one case had pressure sores in the RA group while two cases developed dural tears in the FH group. No revision was required in both groups.

**Conclusion:**

Combined with other surgical correction modalities, robot-assisted pedicle screw fixation is an effective and safe method of treating degenerative scoliosis. Due to its satisfactory surgical outcomes such as higher accuracy and less trauma, it provides a good alternative for clinical practice.

**Level of evidence:**

3.

## Introduction

Adult degenerative scoliosis, also called adult “De Novo” scoliosis, is characterized by a spinal column deformity (Cobb angle> 10° in coronal plain) due to osteoporosis and progressive degeneration of spinal apparatus such as facet joint arthrosis and disc degeneration [1–3]. Studies showed that surgical treatment could improve the mid-long-term quality of life in symptomatic patients [4]. Among them, pedicle screw fixation plays an important role in the correction of deformity [5]. However, adverse factors such as osteoporosis, spinal stenosis, severe degeneration of facet joints, pedicle rotation, and sclerosis have greatly increased the difficulty of screw placement. Besides, incidence of postoperative complications such as internal fixation failure, non-union, and proximal junction kyphosis (PJK) are still relatively high [6]. Therefore, in order to reduce the unfavorable factors such as the loosening of the trajectory, deformity surgery requires extremely high surgical skills from doctors, who should strive for a successful screw placement at the first attempt.

In recent years, computer-assisted navigation and robotic techniques (e.g., Renaissance, ROSA, and TIANJI) have been introduced to spinal surgery, which has stepped to a new stage [7–10]. Studies have showed that robot-assisted spine surgery could improve the accuracy of screw placement and reduce radiation exposure [11]. The potential value of robotic surgery might be greater when it comes to situation such as complex anatomical structures (e.g., cervical spine and spinal deformities) [12]. For spinal deformity, several studies have proved the superior accuracy and perioperative outcomes of robot-assisted pedicle screw placement for adolescent idiopathic scoliosis [13–17]. However, few retrospective studies have focused on the application of robotic surgery to adult degenerative scoliosis [18, 19]. Moreover, only the radiographic accuracy of screw placement and perioperative outcomes were recorded and compared while the follow-up data such as SRS-22 and ODI scores were absent in these studies. Therefore, the purpose of this study was to compare the accuracy and short-term clinical outcomes of pedicle screw placement between robotic-assisted (RA) and freehand (FH) technique in the treatment of adult degenerative scoliosis (ADS).

## Materials and methods

### Subjects

Retrospective study approval was obtained from the institutional review board of our hospital. All patients have written the informed consent. The inclusion criteria were as follows: (1) degenerative scoliosis continuously evolved on the basis of precedent scoliosis with imbalance in the sagittal or coronal planes, which required screw fixation; (2) age ranging from 45 to 80 years old; and (3) patients complied with study. Patients were excluded according to these criteria: (1) degenerative scoliosis with infection, tumor, tuberculosis, and active fracture; (2) history of spinal surgery on intended level; (3) severe preoperative comorbidities; and (4) incomplete data for reviewing the case. Based on the time of introducing the robotic device (December 2018), consecutive patients were assigned into the robot-assisted pedicle screw placement group (RA group) and freehand pedicle screw placement group (FH group) from February 2018 to October 2019. Patient demographics were recorded and compared between two groups. Gertzbein-Robbins grading system was adopted to evaluate the accuracy of pedicle screw placement by means of postoperative CT scan. Short-term clinical outcomes consist of operative time, intraoperative blood loss, length of hospital stay (LOS), radiological parameters, Scoliosis Research Society-22 (SRS-22) score before the operation and 6 months after operation, adverse events, and revisions, which are reviewed between groups. All surgeries were performed by a same team led by a senior orthopedic spine surgeon (> 100 robot-assisted spinal surgeries/year) with 30 years of clinical experience.

### RA procedure

In the robot-assisted group, the surgical team used the novel TIANJI Robot system (TINAVI Medical Technologies Co., Ltd., Beijing, China) to conduct open robot-assisted posterior deformity correction procedure. Neuromonitoring was used in all cases to detect potential neurological injury. Patients were placed on the radio-transparent operating table (Mizuho OSI, USA) at prone position after general anesthesia. The patient tracker was placed on the spinal process, and the calibrator was placed as close as possible to the surgical segment. 3D radiographic images were obtained by the 3D C-arm scanner (Siemens, Germany), and images were transferred to the workstation. After that, surgeons could make plans for the pedicle screw trajectories on the workstation, in sagittal, coronal, and axial views. Then, guide wires and pedicle screws were drilled by hand under the guidance of TIANJI Robot, which had a guidance tube on its arm. During the placement of pedicle screws, the TIANJI Robot system could provide real-time monitoring and adjustment (Figs. 2 and 3). If necessary, the procedure was followed by decompression, posterior osteotomy, rod fixation, and interbody cage insertion for correction and fixation.

### FH procedure

In the conventional freehand group, patients were placed on the radio-transparent operating table at prone position after general anesthesia. Also, neuromonitoring was used in all cases. Open posterior deformity correction procedure was performed through a midline incision on the back. After identifying the anatomical landmarks for screw entry points, pedicle screws were drilled by hand and verified by fluoroscopy. Intraoperative 3D C-arm scans were performed after pedicle screw placement. Decompression, fusion, and posterior osteotomy could be performed subsequently for better scoliosis correction.

### Accuracy measurements

The position of screw placement was assessed according to the Gertzbein-Robbins grading system, which utilized coronal, sagittal, and axial reconstruction views of CT scan [20]. The classifications were as follows: Grade A, screw is completely within the pedicle; grade B, pedicle cortical breach < 2 mm; grade C, pedicle cortical breach < 4 mm; grade D, pedicle cortical breach < 6 mm; and grade E, pedicle cortical breach > 6 mm. Clinically, acceptable screws was defined as grade A and B screws. These measurements were conducted by two separate spine surgeons outside the study in a blinded fashion. Each of spine surgeons owns a MD degree and has at least 5 years of clinical experience. Once the disagreement occurred, two surgeons would re-evaluate the CTs together and analyzed it again after a minimum interval of 1 week to reach the final consensus.

### Outcome measurements

Operative time, intraoperative blood loss, length of stay, adverse events (e.g., pedicle screw loosening, and rod breakage), and revisions were recorded. Standing posteroanterior and lateral radiographs were obtained preoperatively and postoperatively, which are also evaluated by the two independent spine surgeons mentioned above in a blinded way. Radiological parameters include primary curve Cobb angle, apical vertebral translation (AVT), pelvic incidence (PI), lumbar lordosis (LL), and sagittal vertical axis (SVA). Average value was obtained from measurement of two independent observers. SRS-22 (Scoliosis Research Society-22) scores were collected during inpatient and outpatient follow-up on first day pre-operatively and 6 months post-operatively by the same surgical team. SRS-22 is a spinal deformity-specific questionnaire which has 22 items and 5 domains: pain, activity, appearance, mental, and satisfaction. Each domain score ranges from 1 to 5, with higher scores indicating better outcomes. Here, we calculated each domain (pain, function, self-image, mental health, and satisfaction) and total score for patient-reported outcome measurement.

### Statistical analysis

The SPSS 25.0 software was used for the statistical analysis. The G*Power 3.1 software was applied in power analysis. Measurement data is expressed by $\overline{\mathrm{x}}$±s. Independent sample *t* tests were used for measurement data outcome comparison. Paired-sample *t* tests were used to compare the changes between preoperative and postoperative outcomes measurements. Differences of enumeration data were compared using chi-square tests. Cochran-Mantel-Haenszel-chi-square tests and z-test (Bonferroni method) were used for accuracy measurements. According to the existing background information and available samples, expected effect size (d) was set at 0.8, and power (1-β) was set at 0.95. After calculation, *α* was set at 0.046.

## Results

### Demographic characteristics

**Fig. 1:**
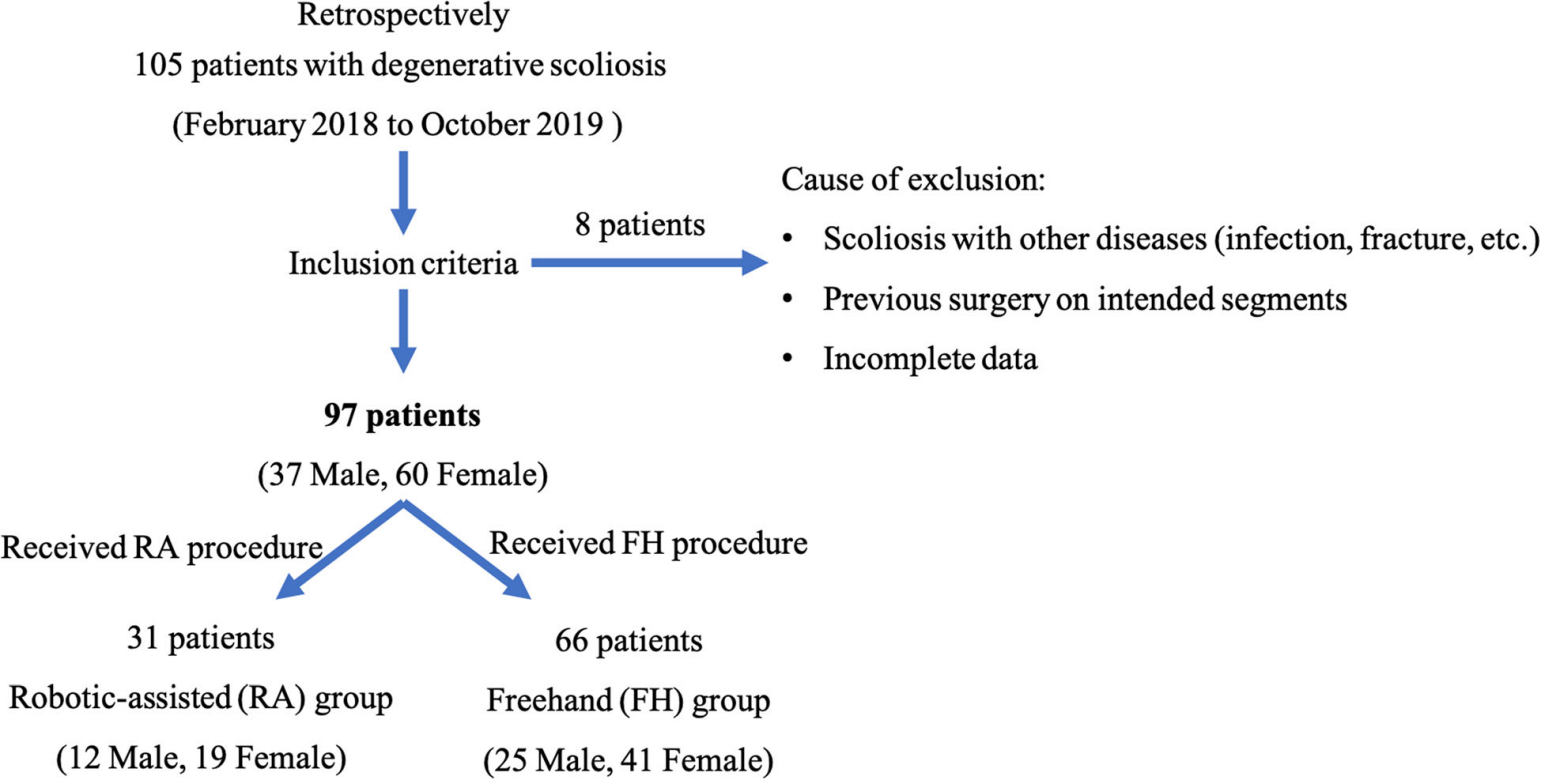
Flow chart showing process of patient selection and exclusion

**Fig. 2:**
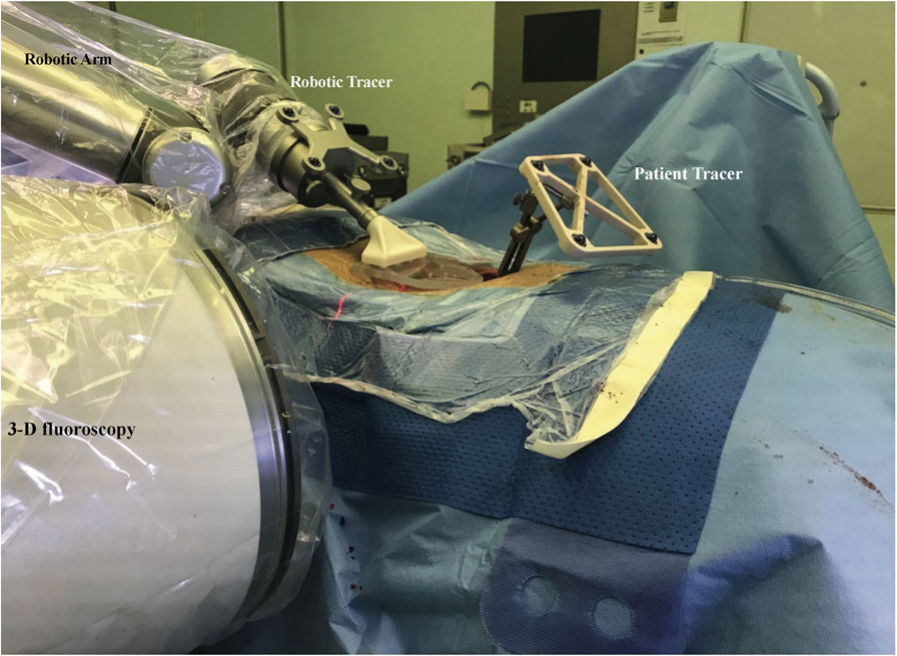
The intraoperative 3D fluoroscopy of robot-assisted spine surgery.

From February 2018 to October 2019, 97 adult patients with degenerative scoliosis admitted to our department were retrospectively reviewed. Thirty-one patients (12 male and 19 female) received robot-assisted pedicle screw placement (RA group), and 66 patients (25 male and 41 female) underwent freehand pedicle screw placement (FH group). The process of patient selection was shown in Fig. 1. Table 1 showed patient demographics of two groups. The mean patient age was 69.5 ± 4.7 years, ranging from 48 to 79. The mean BMI was 24.6 ± 2.1 kg/m^2^. According to the SRS-Schwab classification, type L was accounted for 65.98% (*n* = 64), type T for 23.71% (*n* = 23), type D for 7.22% (*n* = 7), and type N for 3.09% (*n* = 3). According to the Nash-Moe classification at the apex vertebrae of the primary curve, grade I was accounted for 65 cases, grade II for 21 cases, grade III for 8 cases, and grade IV for 3 cases. The average number of screws inserted per case was 12.0 ± 2.5. The average number of fixed segments was 6.2 ± 1.3, ranging from 3 to 11 segments. All clinical details for these 97 patients are shown in Table 1. The mean follow-up time was 11.1 months (range from 6 to 24 months) (Fig. 2).

**Table 1:** Patient demographics in two groups

Characteristics	RA group	FH group	***P*** value
	*N* = 31	*N* = 66	
Age (yrs)	69.8 ± 3.8	69.3 ± 5.1	0.66
Sex (M/F)	12/19	25/41	0.94
Mean BMI (kg/m^2^)	24.5 ± 1.9	24.5 ± 2.1	0.89
**Type of scoliosis (SRS classification)**			0.92
Type L	20	44	
Type T	7	16	
Type D	3	4	
Type N	1	2	
**Nash-Moe classification**			0.49
Grade 1	18	47	
Grade 2	8	13	
Grade 3	4	4	
Grade 4	1	2	
**Mean no. of fixed segment**	6.2 ± 1.4	6.2 ± 1.3	0.93
**Screw parameter**			
Total number of screws	378	786	
Average number of screws/case	12.2 ± 2.6	11.9 ± 2.4	0.60

*RA group* robot-assisted group, *FH group* freehand group, *BMI* body mass index, SRS classification: *type L* TL/lumbar only with thoracic curve < 30°, *type T* thoracic only with lumbar curve < 30°, *type D* double curve with at least one T and one TL/L both > 30°, *type N* bo coronal curve, all coronal curves < 30°. Nash-Moe classification: *grade 1* the pedicle in the concave side starts disappearing, *grade 2* the pedicle disappears, *grade 3* the contralateral pedicle (pedicle in the convex side) is in the midline of the vertebra, *grade 4* the contralateral pedicle crosses the midline of the vertebra

### Accuracy measurements

**Fig. 3.**
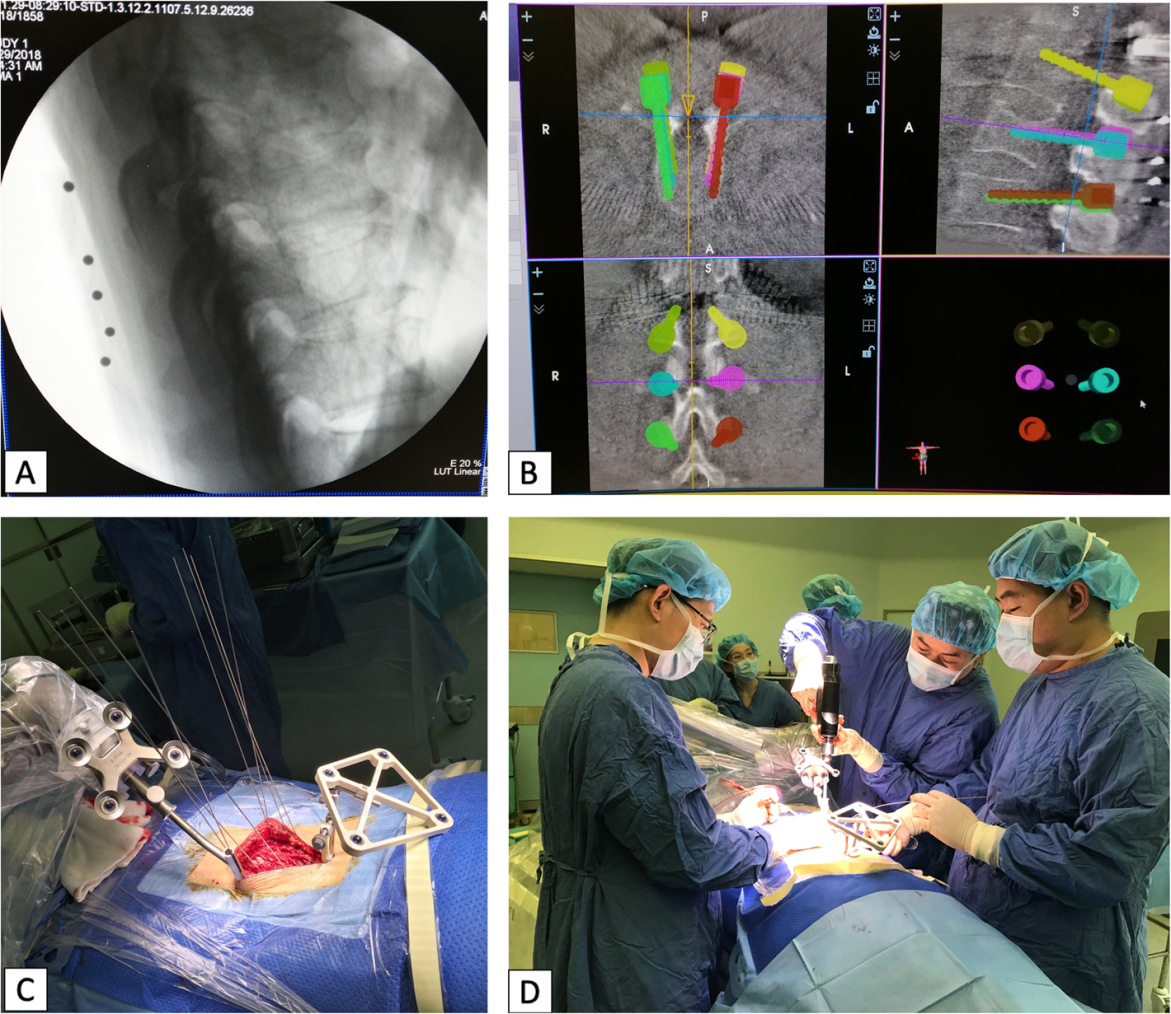
The general operation steps of robot-assisted spine surgery. (1) Registration (**a**), (2) motion planning (**b**), (3) automatic location (**c**), and (4) screw fixation (**d**)

Accuracy measurements of the pedicle screw fixation are summarized in Table 2. In the RA group, 340 (total 378) screws were grade A (89.9%); 33, 4, and 1 were grade B (8.7%), C (1.1%), and D (0.3%). While 589 (total 786) screws achieved grade A (74.9%), 136, 52, and 9 screws were scored as grade B (17.3%), C (6.6%), and D (1.1%). The rate of “perfect” screw position (grade A) in the RA group was higher than that in the FH group (89.9% vs. 74.9%). Moreover, in the RA group, 373 screws were “clinically acceptable” (grade A + B), which was also superior to that in the FH group (98.7% vs. 92.2%, *χ*^2^ = 19.780; *P*< 0.001) (Fig. 3).

**Table 2:** Accuracy measurements of the pedicle screw fixation

Screw grade	RA group (***n***, [%])	FH group (***n***, [%])	***χ***^**2**^ = 38.816, ***p*** < 0.001 ***Adjust P*** value(***z*** test)
A	340 (89.9)	589 (74.9)	< 0.05
B	33 (8.7)	136 (17.3)	< 0.05
C	4 (1.1)	52 (6.6)	< 0.05
D	1 (0.3)	9 (1.1)	
E	0 (0.0)	0 (0.0)	
A + B*	373 (98.7)	725 (92.2)	*χ*^2^ = 19.780; *P*< 0.001

Gertzbein-Robbins grading system: *grade A* screw is completely within the pedicle, *grade B* pedicle cortical breach < 2 mm, *grade C* pedicle cortical breach < 4 mm, *grade D* pedicle cortical breach < 6 mm, *grade E* pedicle cortical breach > 6 mm

*Means the RA group showed superior accuracy in “clinically acceptable” screws

### Surgical outcomes

Intraoperative blood loss of the RA group was less than those in the FH group (499 vs. 573 ml; *P*< 0.001). Operative time (283.1 vs. 291.9 min; *P* = 0.31) and length of stay (12.8 vs. 13.7 days; *P* = 0.36) were compared between RA and FH groups. For patient-reported outcomes, four domains (except satisfaction domain) of the SRS-22 scores at 6 months after operation from both groups were better than those before operation. As for surgery-related complication, one case had pressure sores in the RA group while two cases developed dural tears in thr FH group. No revision was required in both groups. The results of comparisons of surgical outcomes between the two groups are summarized in Tables 3 and 4.

**Table 3:** Comparison of surgical outcomes between the two groups

	RA Group	FH Group	***P*** value
Operative time (min)	283.1 ± 30.8	291.9 ± 40.0	0.31
Intraoperative blood loss (ml)*	498.7 ± 96.3	573.0 ± 78.1	**< 0.001**
Length of hospital stay (day)	12.8 ± 4.5	13.7 ± 4.6	0.36
Perioperative complication	1	2	1.000
Revision	0	0	1.000
**Radiological parameter**			
Cobb (°)			
Pre-op	48.7 ± 6.4	47.4 ± 5.8	0.31
Post-op	10.6 ± 1.9	10.9 ± 1.9	0.39
Correction rate (%)	77.9 ± 5.3	76.6 ± 5.1	0.24
SVA (mm)			
Pre-op	74.3 ± 56.2	79.8 ± 62.5	0.68
Post-op	31.8 ± 20.7	36.3 ± 30.6	0.47
AVT (mm)			
Pre-op	35.6 ± 11.4	39.2 ± 13.4	0.21
Post-op	20.2 ± 6.4	20.7 ± 8.2	0.78
PI (°)			
Pre-op	51.8 ± 3.9	51.2 ± 4.0	0.50
Post-op	50.8 ± 3.4	51.1 ± 3.7	0.69
LL (°)			
Pre-op	22.4 ± 5.4	21.7 ± 6.0	0.62
Post-op	51.8 ± 4.4	50.3 ± 5.0	0.18
**Δ**	29.4 ± 5.4	28.6 ± 8.3	0.62
Follow-up period (month)	11.1 ± 4.1	11.1 ± 3.4	0.96

*SVA* sagittal vertical axis, *AVT* apical vertebral translation, *PI* pelvic incidence, *LL* lumbar lordosis, ***Δ****=|* post-pre |

*Means RA group showed less intraoperative blood loss compared with FH group

**Table 4:** Score of SRS-22 for degenerative scoliosis patient from RA group and FH group

SRS-22 domains	Pre-op	6 months post-op*
**Pain**		
RA	2.3 ± 0.7	3.8 ± 1.2
FH	2.5 ± 0.8	3.5 ± 1.0
**Function/activity**		
RA	2.2 ± 0.7	3.5 ± 0.7
FH	2.2 ± 0.6	3.2 ± 0.8
**Self-image/appearance**		
RA	2.3 ± 0.8	3.3 ± 0.9
FH	2.2 ± 0.8	3.3 ± 0.8
**Mental health**		
RA	2.5 ± 0.9	3.6 ± 0.9
FH	2.7 ± 0.9	3.5 ± 0.9
**Satisfaction with management**		
RA	**/**	3.3 ± 0.9
FH	**/**	3.4 ± 0.9
**Total**		
RA	2.3 ± 0.4	3.5 ± 0.4
FH	2.4 ± 0.4	3.4 ± 0.4

*SRS-22* Scoliosis Research Society-22

*Post-op scores were improved compared with pre-op scores from both groups

**Fig. 4:**
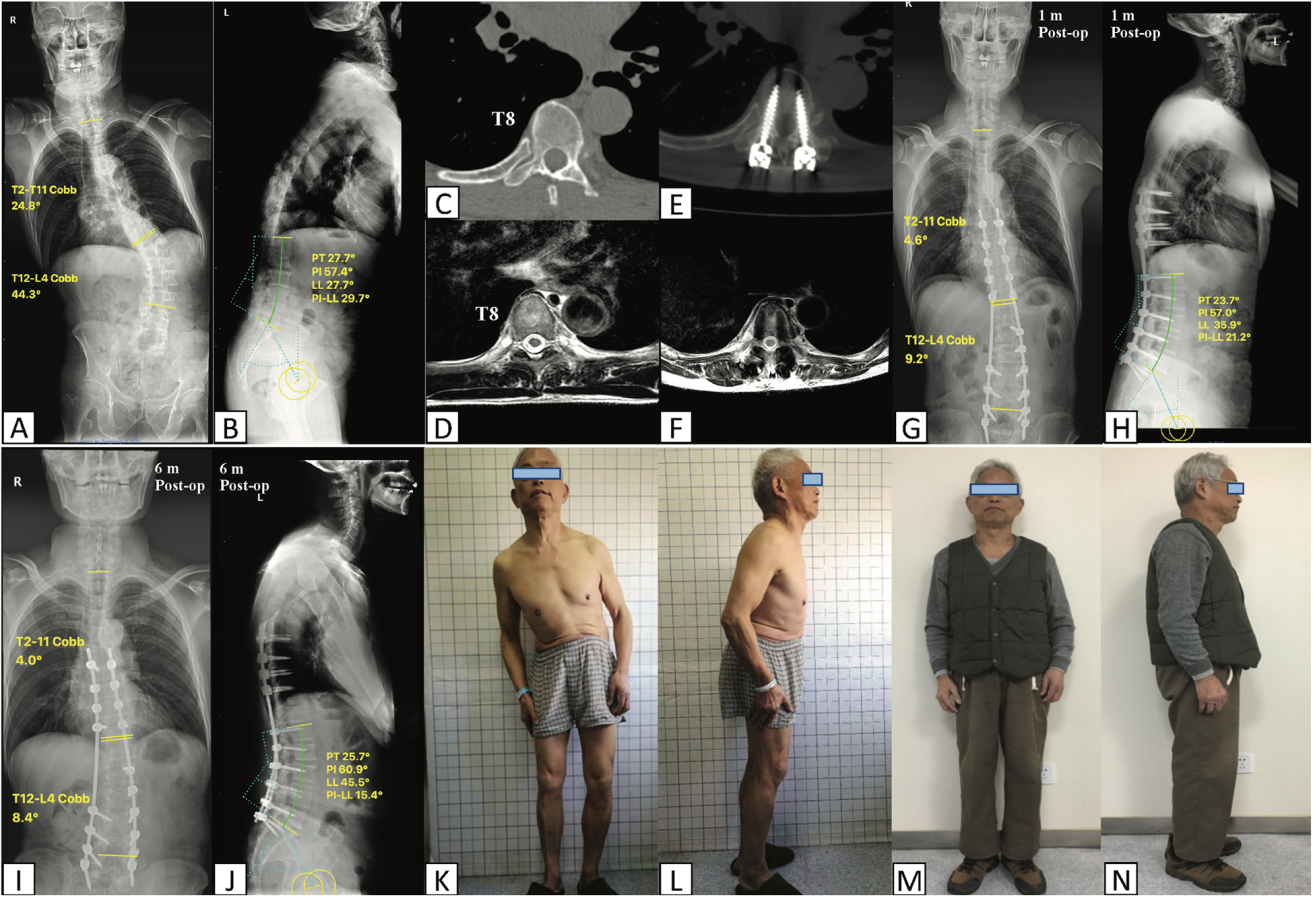
A 74-year-old gentleman with spine deformity for 3 years (K-L). The radiographs demonstrate a lumbar curve of 44.3° and a thoracic curve of 24.8° (**a**). The sagittal parameters were shown in (**b**). Postoperative 1 month and 6 months posteroanterior and lateral radiographs demonstrate satisfying correction of degenerative scoliosis (g–j, m, n). **c**, **d** Axial plane of pre-op CT and MRI. **e**, **f** Post-op CT and MRI revealed satisfying position of T8 pedicle screws (grade A)

**Fig. 5:**
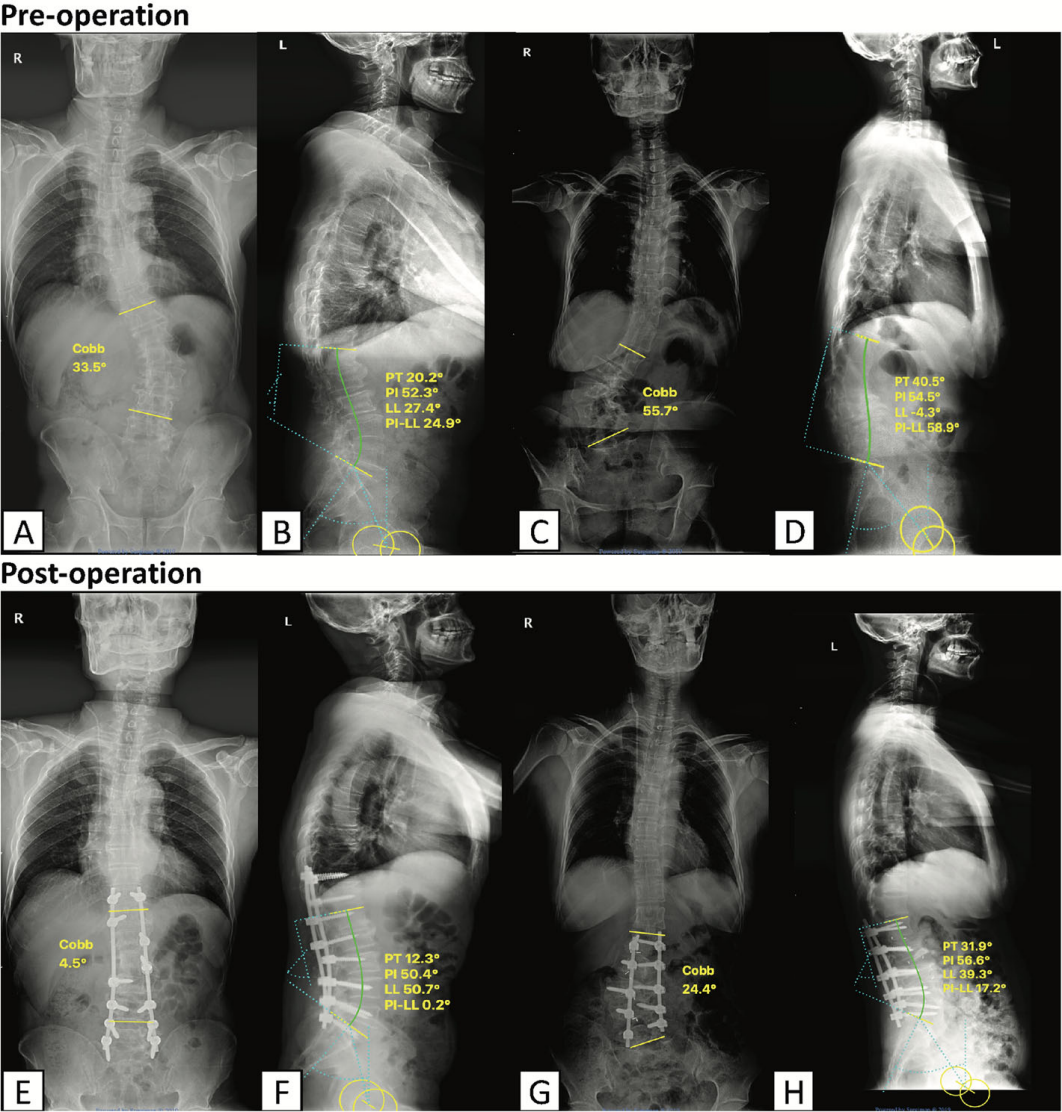
Pre-operative (**a**–**d**) and post-operative (**e**–**h**) radiographs of two cases including a 61-year-old male (**a**, **b**, and **e**, **f**) and a 48-year-old female (**c**, **d** and **g**, **h**) with degenerative scoliosis were shown in this figure. Both of them underwent robot-assisted scoliosis correction surgery. The average operative time was 240 min, and mean blood loss was 400 ml. Symptoms including unstable gait and lower limbs numbness improved after surgeries

Also, with regard to radiological parameters such as Cobb angles, SVA, AVT, PI and LL, both of groups were improved postoperatively. Three cases including pre-operative and post-operative radiographs were described in Figs. 4 and 5.

## Discussion

For symptomatic ADS, surgical treatment is often required to correct and restore the imbalance and symptoms through various modalities such as pedicle screw placement with PLIF and osteotomy [21]. However, it is more difficult for surgeon to insert the pedicle screw because of the abnormal anatomical structures of surgical intended vertebra. In the past decades, rapid development of robotic surgery has brought the spinal surgery into a new level. With exquisite robotic assistance, spine surgeons can perform almost all kinds of spinal operation in a more accurate and precise way [8]. The TIANJI Robot system, which we used in this research, is a multi-indication orthopedic surgical robot developed and approved by China in 2016. So far, there was rare literature report about the robotic application in the surgical treatment of ADS. With utilization of TIANJI Robot system, this study compared the short-term clinical outcomes and accuracy of pedicle screw placement between RA and FH technique in the treatment of degenerative scoliosis.

The final outcomes turned out that RA technique was superior to the FH technique in the aspect of screw fixation accuracy and intraoperative blood loss. Several previous studies which focused on scoliosis aligned with the conclusion of higher screw fixation accuracy of the RA group. Fan et al. [18] reported that RA technique showed higher accuracy for screw implantations in degenerative scoliosis. K. Aaron et al. [13] showed that the overall accuracy of robot-assisted pedicle screw insertion was as high as 97% in the treatment of adolescent idiopathic scoliosis (AIS). Also, Jeremy et al. [14] reported an accuracy rate of 97.6 % in the robot-assisted pedicle screw placement in AIS. The overall accuracy rate for robotic-guided S2AI screw placement was 95.7% in elderly degenerative spinal deformities, according to Joseph et al. [15]. However, only limited studies evaluated the accuracy of RA pedicle screw placement in elderly population, which was the driving force for us to conduct this study.

On the other hand, there were several studies which were skeptical of the higher accuracy of the RA technique. In a randomized controlled trial conducted by Ringel et al. [22] in 2012, poorer screw placement was found in the RA group (85% vs. 93%). To date, a consensus on the higher accuracy of RA technique for the degenerative scoliosis has not been reached yet. We consider that some factors may contribute to this controversy. One is the various robotic models designed by different robotic manufacturers. The other may be the inadequate training of the surgeon due to learning curve of robotic surgery. However, more recent studies have proved the higher accuracy of RA screw placement [23, 24]. In a randomized controlled trial conducted by Fan et al. [12] in 2019, improved accuracy and clinical outcomes were reported in cervical RA spinal surgery (98.9% vs. 91.2%). A study found that the use of RA techniques can improve the accuracy of screw placement between residents and attending surgeons (97.67% vs. 98.67%) [25]. This shows that RA technique not only has a short learning curve, but also can provide homogeneous treatment for patients delivered by trained doctors. What’s more, it has been reported in the literature that RA screw placement errors are most likely to occur in two stages: 10 to 20 cases and about 40 cases [26]. Therefore, the researchers suggest that novices should perform the RA surgery under strict supervision and guidance in the first 25 cases of robotic surgery. We believe that, with constant improvement and modification of robotic designs, the higher accuracy of RA technique in pedicle screw fixation will be generally recognized in the future.

Speaking of the surgical outcomes, intraoperative blood loss was less in the RA group. This could be attributed to less surgical exposure and higher accuracy of the RA navigation and operation which might lead to less chance of intraoperative screw revisions. For other outcomes parameters, although the RA technique could provide higher accuracy and less blood loss, they seemed to have similar operative time between two groups. In our opinion, although RA technique might help simplify and shorten the period of screw placement, the duration of whole operation could be expanded because of the additional intraoperative planning and preparation time. Length of hospital stay in the RA group was slightly shorter than that of the FH group with a *P* value of 0.36. We considered this might attribute to the small sample size of the RA group. For both groups, the SRS-22 scores at 6 months after operation were better than those before operation. The SRS-22 score can effectively evaluate health-related quality of life in patients with degenerative scoliosis, which is even more responsive than Oswestry Disability Index (ODI) [27, 28]. The improvement of SRS-22 implied that both techniques could improve pain, self-image, and functional status of the patient. One case with pressure sores occurred in the RA group while two cases developed dural tears in the FH group. No revision was required in both groups. Considering the short time of follow-up, this result may change with time. In the beginning, we were curious about the possible relation between the screw accuracy and scoliosis correction. The results turned out that different accuracy between two groups did not certainly affect difference in correction parameters. Both groups showed improved radiological spinal alignment after the operation.

Till now, the robot-assisted surgery still has several limitations. In practice, we found that it was difficult to complete a 360° scan of the severe kyphosis patient intraoperatively, because the 3D C-arm scanner had a limited circumferential diameter. Secondly, for long-segment deformity surgery, multiple scans and planning were often required, which might increase the operative time and radiation exposure. At present, robot plays its role mainly in the placement of pedicle screws, while it cannot provide effective assistance for surgical procedure such as osteotomy, decompression, or three-dimensional correction of deformities. Therefore, improvement measures should be carried out to solve these problems such as function extension and sharing radiological information between the robot and picture archiving and communication systems (PACS) of the hospital.

This study had its inherent limitations. First, it was a retrospective study with small sample size of the RA group. In this preliminary clinical report, although we tried to minimize some potential confounding factors such as different learning curves, other serious confounders (i.e., confounding by indication, non-randomization, and different surgical strategies) could not be taken into consideration. Thus, further study with randomized control and larger number of patients is warranted. What’s more, the follow-up time is short which requires further follow-up. Second, radiation exposure to the medical staff and patient was absent, which is also an important aspect to compare. Third, given the financial burden from the robotic surgery and long learning curve, to popularize and promote the robotic technique remains a challenge.

## Conclusion

Combined with other surgical correction modalities, robot-assisted pedicle screw fixation is an effective and safe method of treating degenerative scoliosis. Due to its satisfactory surgical outcomes such as higher accuracy and less trauma, it provides a good alternative for clinical practice.

## Data Availability

The datasets used and/or analyzed during the current study are available from the corresponding author on reasonable request.

## Abbreviations

ADS: Adult degenerative scoliosis
SRS-22: Scoliosis Research Society-22
AVT: Apical vertebral translation (AVT)
PI: Pelvic incidence
LL: Lumbar lordosis
SVA: Sagittal vertical axis

## References

[1] Ploumis A, Transfledt EE, Denis F (2007). Degenerative lumbar scoliosis associated with spinal stenosis. Spine J.

[2] Glassman SD, Bridwell K, Dimar JR, Horton W, Berven S, Schwab F (2005). The impact of positive sagittal balance in adult spinal deformity. Spine (Phila Pa 1976).

[3] Schwab FJ, Smith VA, Biserni M, Gamez L, Farcy JP, Pagala M (2002). Adult scoliosis: a quantitative radiographic and clinical analysis. Spine (Phila Pa 1976).

[4] Kyrölä K, Kautiainen H, Pekkanen L, Mäkelä P, Kiviranta I, Häkkinen A (2019). Long-term clinical and radiographic outcomes and patient satisfaction after adult spinal deformity correction. Scand J Surg.

[5] Chen PG, Daubs MD, Berven S, Raaen LB, Anderson AT, Asch SM (2016). Surgery for degenerative lumbar scoliosis: the development of appropriateness criteria. Spine (Phila Pa 1976).

[6] Charles YP, Ntilikina Y (2019). Scoliosis surgery in adulthood: what challenges for what outcome?. Ann Translat Med.

[7] Joseph JR, Smith BW, Liu X, Park P (2017). Current applications of robotics in spine surgery: a systematic review of the literature. Neurosurg Focus.

[8] Overley SC, Cho SK, Mehta AI, Arnold PM (2017). Navigation and robotics in spinal surgery: where are we now?. Neurosurgery..

[9] Ghasem A, Sharma A, Greif DN, Alam M, Maaieh MA (2018). The arrival of robotics in spine surgery: a review of the literature. Spine (Phila Pa 1976).

[10] Huang J, Li Y, Huang L (2020). Spine surgical robotics: review of the current application and disadvantages for future perspectives. J Robot Surg.

[11] Kochanski RB, Lombardi JM, Laratta JL, Lehman RA, O'Toole JE (2019). Image-guided navigation and robotics in spine surgery. Neurosurgery..

[12] Fan M, Liu Y, He D, Han X, Zhao J, Duan F (2020). Improved accuracy of cervical spinal surgery with robot-assisted screw insertion: a prospective, randomized, controlled study. Spine..

[13] Shaw KA, Murphy JS, Devito DP (2018). Accuracy of robot-assisted pedicle screw insertion in adolescent idiopathic scoliosis: is triggered electromyographic pedicle screw stimulation necessary?. J Spine Surg.

[14] Macke JJ, Woo R, Varich L (2016). Accuracy of robot-assisted pedicle screw placement for adolescent idiopathic scoliosis in the pediatric population. J Robot Surg.

[15] Laratta JL, Shillingford JN, Lombardi JM, Alrabaa RG, Benkli B, Fischer C (2018). Accuracy of S2 alar-iliac screw placement under robotic guidance. Spine Deform.

[16] Ughwanogho E, Patel NM, Baldwin KD, Sampson NR, Flynn JM (2012). Computed tomography-guided navigation of thoracic pedicle screws for adolescent idiopathic scoliosis results in more accurate placement and less screw removal. Spine (Phila Pa 1976).

[17] Chan A, Parent E, Narvacan K, San C, Lou E (2017). Intraoperative image guidance compared with free-hand methods in adolescent idiopathic scoliosis posterior spinal surgery: a systematic review on screw-related complications and breach rates. Spine J.

[18] Fan Y, Peng Du J, Liu JJ, Zhang JN, Liu SC, Hao DJ (2018). Radiological and clinical differences among three assisted technologies in pedicle screw fixation of adult degenerative scoliosis. Sci Rep.

[19] Hyun SJ, Kim KJ, Jahng TA (2017). S2 alar iliac screw placement under robotic guidance for adult spinal deformity patients: technical note. Eur Spine J.

[20] van Dijk JD, van den Ende RP, Stramigioli S, Kochling M, Hoss N (2015). Clinical pedicle screw accuracy and deviation from planning in robot-guided spine surgery: robot-guided pedicle screw accuracy. Spine (Phila Pa 1976).

[21] Simmons ED (2001). Surgical treatment of patients with lumbar spinal stenosis with associated scoliosis. Clin Orthop Relat Res.

[22] Ringel F, Stuer C, Reinke A, Preuss A, Behr M, Auer F (2012). Accuracy of robot-assisted placement of lumbar and sacral pedicle screws: a prospective randomized comparison to conventional freehand screw implantation. Spine (Phila Pa 1976).

[23] Han X, Tian W, Liu Y, Liu B, He D, Sun Y, et al. Safety and accuracy of robot-assisted versus fluoroscopy-assisted pedicle screw insertion in thoracolumbar spinal surgery: a prospective randomized controlled trial. J Neurosurg Spine. 2019:1–8. 10.3171/2018.10.SPINE18487 30738398

[24] Li HM, Zhang RJ, Shen CL (2020). Accuracy of pedicle screw placement and clinical outcomes of robot-assisted technique versus conventional freehand technique in spine surgery from nine randomized controlled trials: a meta-analysis. Spine (Phila Pa 1976).

[25] Vardiman AB, Wallace DJ, Booher GA, Crawford NR, Riggleman JR, Greeley SL, et al. Does the accuracy of pedicle screw placement differ between the attending surgeon and resident in navigated robotic-assisted minimally invasive spine surgery? J Robot Surg. 2019. 10.1007/s11701-019-01019-9. 10.1007/s11701-019-01019-9 PMC7347677 31542860

[26] Schatlo B, Martinez R, Alaid A, von Eckardstein K, Akhavan-Sigari R, Hahn A (2015). Unskilled unawareness and the learning curve in robotic spine surgery. Acta Neurochir.

[27] Bridwell KH, Berven S, Glassman S, Hamill C, Horton WC, Lenke LG (2007). Is the SRS-22 instrument responsive to change in adult scoliosis patients having primary spinal deformity surgery?. Spine (Phila Pa 1976).

[28] Bridwell KH, Cats-Baril W, Harrast J, Berven S, Glassman S, Farcy JP (2005). The validity of the SRS-22 instrument in an adult spinal deformity population compared with the Oswestry and SF-12: a study of response distribution, concurrent validity, internal consistency, and reliability. Spine (Phila Pa 1976).

